# Navigating in foldonia: Using accelerated molecular dynamics to explore stability, unfolding and self-healing of the β-solenoid structure formed by a silk-like polypeptide

**DOI:** 10.1371/journal.pcbi.1005446

**Published:** 2017-03-22

**Authors:** Binwu Zhao, Martien A. Cohen Stuart, Carol K. Hall

**Affiliations:** 1 Department of Chemical and Biomolecular Engineering, North Carolina State University, Raleigh, North Carolina, United States; 2 Laboratory of Physical Chemistry & Colloid Science, Wageningen University, NL, Wageningen, The Netherlands; Fudan University, CHINA

## Abstract

The β roll molecules with sequence (GAGAGAGQ)_10_ stack via hydrogen bonding to form fibrils which have been themselves been used to make viral capsids of DNA strands, supramolecular nanotapes and pH-responsive gels. Accelerated molecular dynamics (aMD) simulations are used to investigate the unfolding of a stack of two β roll molecules, (GAGAGAGQ)_10_, to shed light on the folding mechanism by which silk-inspired polypeptides form fibrils and to identify the dominant forces that keep the silk-inspired polypeptide in a β roll configuration. Our study shows that a molecule in a stack of two β roll molecules unfolds in a step-wise fashion mainly from the C terminal. The bottom template is found to play an important role in stabilizing the β roll structure of the molecule on top by strengthening the hydrogen bonds in the layer that it contacts. Vertical hydrogen bonds within the β roll structure are considerably weaker than lateral hydrogen bonds, signifying the importance of lateral hydrogen bonds in stabilizing the β roll structure. Finally, an intermediate structure was found containing a β hairpin and an anti-parallel β sheet consisting of strands from the top and bottom molecules, revealing the self-healing ability of the β roll stack.

## Author summary

Silk-inspired repeated sequences, variants of the sequence from Bombyx Mori silk, have been used to make supramolecular nanotapes, pH-responsive gels, and most importantly self-assembled coat for artificial viruses. Silk-inspired repeated sequences have shown great potential as promising delivery vehicles in targeted delivery of nucleic acids for gene therapy. However, the mechanisms regarding the folding and docking of silk-inspired polypeptides remain elusive. Elucidation of the folding and docking mechanism might help us create sequences with desired self-assembly properties for many biomedical applications. An enhance sampling method, accelerated molecular dynamics (aMD) simulation, is used in this study to investigate the unfolding of a stack of two β roll molecules to shed light on the folding mechanism by which silk-inspired polypeptides form fibrils and to identify the dominant forces that keep the silk-inspired polypeptide in a β roll configuration. A template-unfolding mechanism and a neat step-wise unfolding fashion are found which agree well with experimental observations.

## Introduction

An increasing number of functional proteins are reported to have β solenoid structure, such as antifreeze protein [1–3], curli [4], and carbonic anhydrase enzyme [5]. Formed by winding the peptide chain in a left-handed or right-handed fashion, a β solenoid structure usually has a repeat unit consisting of 2, 3, or 4 β strands that are connected by turns [6]. Each of these strands form parallel β sheets with their neighboring strands. As a result, a β solenoid structure usually has two parallel β sheets with the strands facing in different directions. The interior of β solenoid structures contains apolar amino acid side chains that are tightly packed [6], sometimes even interdigitated [7], resulting in a predominately hydrophobic core structure.

Polypeptides in β solenoid structures have been used as building blocks of fibrils via controlled self-assembly in biomaterials applications. They have been used to create viral capsids of DNA strands[8], supramolecular nanotapes [9] and pH-responsive gels [10], which find biomedical application as matrices for human cells [11], Beta solenoids (for short: beta rolls) can form fibrillar structures via two different mechanisms: end-to-end assembly where the terminals are covalently attached to each other, creating a very long β solenoid, or sheet-to-sheet assembly where the sheets associate physically resulting in a stack. Peralta et al. [12] used the former type of self-assembly to generate micron length amyloid fibrils from spruce budworm antifreeze protein, a modified β solenoid protein. Beun et al. used the latter type of self-assembly to produced fibrils made of stacks of pH-responsive silk-collagen-like triblocks [13].

**Fig 1:**
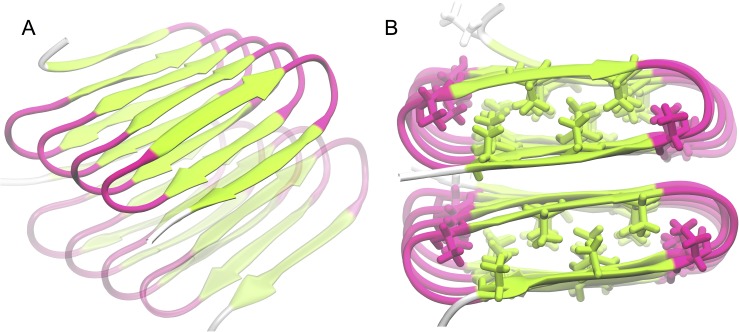
**Top view (A) and a side view (B) of the two-molecule** β **roll stack.** A typical β roll structure has two oppositely oriented β sheets (in green) that are connected by β turns (in pink) and a hydrophobic core with its hydrophobic side chains (as shown in B) buried in the roll.

According to experimental reports, the dimensions of fibrils consisting of stacks of *Bombyx Mori* silk-inspired polypeptides with a sequence of (GAGAGAGX)_n_, where A and G stand for alanine and glycine, respectively, X is a polar residue and n is the number of repeating units [13] are consistent with β-solenoid structures stacked on top of each other [14]. To obtain a better understanding of the detailed structure of these fibrils, we recently investigated the β-solenoid structure formed by (GAGAGAGQ)_10_ via conventional molecular dynamics (cMD) simulations and found that the most probable structure formed by this sequence is a β-roll structure, with all the hydrophobic alanine side chains pointing inward, as shown in Fig 1 [7]. This structure was found to be more stable than a structure reported earlier by Schor et al. where all the hydrophobic alanine side chains pointed outwards [14]. Now that the ‘ground state’ structure of the building block in the filament has been determined, we wish to learn more about how initially disordered polypeptides fold into the β-roll structure and assemble to form the filament.

Two different mechanisms have been proposed regarding the folding and docking of silk-inspired polypeptides. The first mechanism, the “template folding” (TF) mechanism, was deduced from the temporal evolution of CD spectra when the polypeptide (GAGAGAGE)_n_ formed fibrils [13]; according to TF the peptide starts to fold into a β roll structure once it attaches to the growing end of a pre-existing filament. The experimental growth rate of filaments is very low (one molecule-per-second) and the fibril formation is irreversible. The second mechanism, “solution folding” (SF), proposed by Schor et al. [15], is based on atomistic simulations, also for (GAGAGAGE); it claims that a polypeptide first folds in solution into a β-roll structure before it docks to the growing end of a fibril via Glu-Glu side chain interactions. Both mechanisms showed up in replica exchange Monte Carlo simulations carried out by Ni et al. [16], of the folding pathways of silk-inspired polypeptides with sequence [EIAIAIAR]_12_ (I is isoleucin and R is Arginine). They found that at low temperature the polypeptide folds into a β-roll configuration before docking to another molecule, but at high temperature it folds after docking to another molecule. It is important to point out here that the folding pathways proposed by Schor *et al*. [15] and by Ni *et al* [16] are based on the β-roll structure predicted by Schor *et al*. [14], which has all the alanine side chains pointing outwards from the β-roll. However, as mentioned earlier, our recent study [7] found that the β-roll structure formed by the silk-inspired polypeptide (GAGAGAGQ)_10_ has a higher probability to have a hydrophobic core than a hydrophobic shell, i.e. all the alanine side chains should point inwards rather than outwards. Therefore, the folding pathway of the more probable hydrophobic core structure still remains elusive.

We use an enhanced sampling method, accelerated molecular dynamics (aMD), to study the unfolding behavior of a two-molecule β roll stack. We choose this method because conventional molecular dynamics (cMD) simulations of the folding of a peptide into a β roll are too slow. This is understandable: the experimental time scale, corresponding to the addition of a single molecule to the growing end of a fibrils is thought to take about a second [13] which is orders of magnitude away from time scales reached in MD simulations. Accelerated molecular dynamics (aMD) has been used on many different oligomers and polypeptides, including alanine dipeptide [17], bovine pancreatic trypsin inhibitor (BPT1) [18], G-protein coupled receptors [19], and streptavidin-biotin complex [20]. One of the advantages of aMD compared to other enhanced sampling methods is that it does not require pre-defined reaction coordinates. This allows simulations to explore a broader range of hypothetical kinetic pathways than would otherwise be possible. In aMD simulations of proteins, boost potentials are added to the potential energy; for proteins one can choose to boost either the total potential energy of the system, or the dihedral energy, or both. The boost potential is only added to potentials below a pre-defined threshold energy; for energies above that level the original shape is retained. aMD makes barrier crossing between low energy states easier and therefore provides access to regions of conformational space that are unreachable in cMD simulations.

The long term goal of our study is to elucidate the folding mechanism of silk-inspired polypeptide, as well to identify the dominant forces that maintain the β roll configuration. As there is no unambiguous way to choose a representative starting configuration, we decided to focus on unfolding rather than folding, which should give us clues as to the likeliness of hypothetical folding pathways. To this end, we simulate a stack of two molecules having sequence (GAGAGAGQ)_10,_ each folded into a β roll with a hydrophobic core (Fig 1) and in explicit solvent. aMD is used to study the unfolding behavior of one of the β roll structures at systematically increased values for the threshold. We first study the system with a stack of two β roll molecules, using the lowest threshold. Then we simulate a system with a partially-fixed bottom template and a relatively high threshold; this enables us to observe the unfolding behavior of a single molecule on top of the template, and also gives us information about which forces are most important in maintaining the β roll structure. Finally, we challenge the system with partially-fixed bottom template by a further increase of the threshold to gain a better picture of the intermediate structures that form along the entire unfolding pathway.

The major findings in this paper are the following. First, we find that in the system without fixed atoms the molecules unfold in a step-wise fashion, and both molecules unfold completely by the end of the simulation. The unfolding is initiated mainly from the C terminal. Second, in the stack with a partially-fixed bottom template the top molecule has more difficulty unfolding than the molecules in the stack without any fixed atoms, indicating the importance of the template in stabilizing the folded structure of a β roll molecule in a stack. Third, there is a hierarchy of hydrogen bond strengths. Lateral hydrogen bonds formed between β strands within the β sheets in a β roll structure are stronger than the vertical hydrogen bonds within the β turns; H bonds are weaker when they are closer to the sides of the β turns. The lateral hydrogen bonds within the bottom layer in the top β roll molecule are stronger than those in the top layer, which suggests that the bottom template strengthens the hydrogen bonds within the β sheet that it contacts. As the aMD threshold on a system with partially-fixed bottom template is further increased, we find that the β roll on top tends to form hydrogen bonds with the bottom template to resist leaving it, revealing a “self-healing” property, which helps explain the toughness of the fibrils formed in the experiment.

## Results

### Molecules in β roll structure unfold in a step-wise fashion

We begin by describing our aMD simulation results on the stack of two β roll molecules with sequence, (GAGAGAGQ)_10_, without fixed atoms, and at the lowest threshold as characterized by *n* = 2 in Eq 7. (Recall that *n* is an integer in Eq 7 that determines the magnitude of the threshold as a multiple of the acceleration factor.)

**Fig 2:**
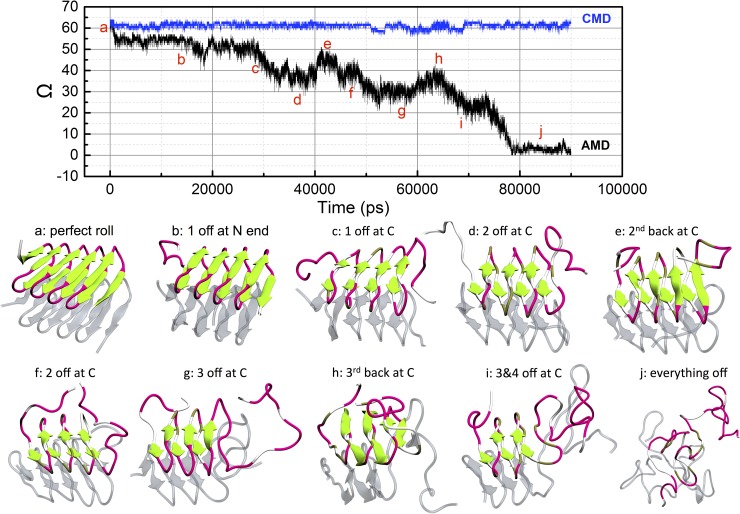
Order parameter, Ω, of the top molecule in the stack of two β roll molecules plotted against simulation time and representative structures at each stage from clustering analysis. The Ω of the top molecule in the cMD simulations (in blue) remains at around 62, and the Ω of the top molecule in the aMD simulations (in black) decreases in a step-wise fashion. The order parameter, Ω, at different stages of the aMD are labeled from a to j. The β roll is in a perfect condition with no strands off (a), then the first strand comes off N terminal (b); another strand comes of C terminal (c), followed by the second strand lifting off from C terminal (d), after which the second strand from C terminal goes back to the β roll (e); then the second strand comes off C terminal (f), followed by the third strand off the C terminal (g); then the third strand from the C terminal goes back (h), after that the third and the fourth strands come off C terminal simultaneously (i); finally the chain unwraps completely (j). The representative structures of each stage generated from clustering analysis are shown below the plot, with β sheets in green, β turns in pink and random coil structures in white. The bottom molecule is silver with a transparent representation.

In Fig 2, the order parameter of the top molecule, Ω, which measures the departure of the β roll from its ideal structure, is plotted against the simulation time, revealing the striking result that molecules in the β roll structure unfold in a step-wise fashion. In cMD simulations, Ω for the top molecule remains roughly constant around 62±2, indicating that the molecule stays in the β roll structure throughout the entire simulation. In contrast, in aMD it decreases in a step-wise fashion and eventually reaches zero. Roughly, five different plateaus can be discerned in the plot: 1–28 ns (Ω = 52), 32–48 ns (Ω = 38), 52–66 ns (Ω = 30), 70–74 ns (Ω = 24), and 78–90 ns (Ω = 2). The representative structures corresponding to the different states shown in Fig 2 were calculated via clustering analysis. The step-wise unfolding of the β roll structure agrees well with single-molecule force spectroscopy (SMFS) measurements by Sapra et al. on the unfolding pathways of β-barrel-forming membrane proteins, OmpG [21]. By mechanically pulling on a single atom at one end of OmpG they found that each β hairpin of the OmpG β barrel unfolded either individually, or cooperatively with an adjacent β hairpin, causing the OmpG protein to unfold in a step-wise fashion.

The unfolding pathway of the top molecule in the stack as revealed by aMD simulation can be described as follows. The molecule starts from a perfect β roll structure (see Fig 2(A) with a Ω of ~ 62 and then reaches its first plateau which lasts from 1 ns to around 28 ns. The representative structure generated from clustering analysis is shown in Fig 2(B) with one strand lifted off from the N terminal. By 28 ns, another strand from the C terminal starts to come off the β roll structure as shown in Fig 2(C). This is a transient state because the order parameter quickly moves to the second plateau that lasts from around 32 ns to 58 ns. The representative structures that occur during this plateau are shown in Fig 2(D), 2(E) and 2(F). The majority of structures that occur during this plateau have one strand off of the C terminal and two strands off of the N terminal as in Fig 2(D) and 2(F). The second strand from the N terminal goes back to the β roll structure for a short period of time within this plateau, from around 41 ns to 44 ns, as shown in Fig 2(E). After 52 ns, the order parameter reaches another plateau, with Ω ~ 30, during which the third strand peels off the β roll from the C terminal as shown in Fig 2(G). This strand goes back to the roll structure for a short period of time as shown in Fig 1(H), and then quickly comes off the β roll together with the fourth strand from the C terminal at around 70 ns, as shown in Fig 2(I). After that, there are only five strands left in the β roll structure, which is not enough to maintain the configuration. Starting at 73 ns, the molecule collapses quickly and becomes a random coil structure. Some refolding events occur during the unfolding process, mainly when one loose strand goes back to its original neighbor. For example, as shown in Fig 2, the second strand comes off the C terminal at stage d, then goes back to the C terminal at stage e, and finally comes off the C terminal again at stage f. Moreover, the third strand from the C terminal comes off at stage g, then goes back at stage h, and eventually comes off of the C terminal with the fourth strand at stage i.

The β roll molecules thus appear to unfold in an asymmetric fashion, namely mainly from the C terminal, as evidenced by the unfolding pathway just described. This finding agrees well with a report by Alsteens et al. based on a steered molecular dynamics (sMD) simulation study in which a prototypic TpsA protein, FHA [22] unfolds mainly from the C terminal. In addition, our simulation suggests that a β roll configuration needs to have a nucleus of a certain size to maintain its structure: the sequence (GAGAGAGQ)_10_ needs to have at least half of its strands, 5 strands, in a β roll structure, in order to maintain the β roll configuration. With less folded strands, it collapses and forms an amorphous configuration.

**Fig 3:**
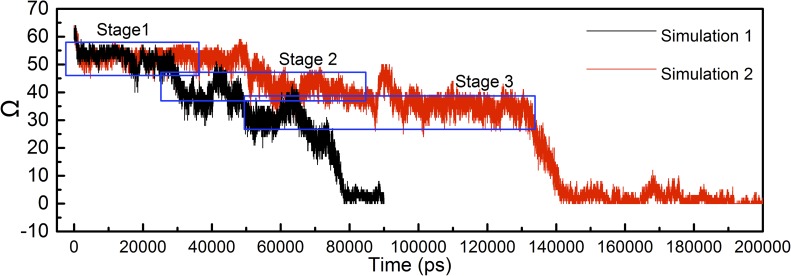
Order parameter, Ω, of the top molecule in the stack of two β roll molecules plotted against simulation time of the simulation in Fig 2 (black) and an additional simulation (red) with the same aMD boost parameter, n = 2. The order parameters of both simulations go through 3 stages (outlined in blue) before reaching zero.

A second simulation of the two-molecule stack without fixed atoms having the same aMD boost parameter (n = 2) was performed in order to check for reproducibility. This second simulation was performed for 200 ns longer than the first simulation as the chain took longer to completely unfold. Both simulations exhibit step-wise unfolding behaviors as can be seen in Fig 3, which plots the order parameter, Ω, versus time for Simulations 1 and 2. The two simulations go through the same stages as the unwrapping occurs, each stage is outlined in blue in Fig 3. This similarity helps to support the reproducibility of our simulations of the unfolding process.

### Partially-fixed bottom template helps stabilize the β roll structure on top of it

aMD simulations are then performed on systems containing a stack of (GAGAGAGQ)_10_ β roll molecules with a partially-fixed bottom template at the threshold potential energy with n = 2 in Eq 7. By having a partially-fixed bottom template (as defined above), molecules in the stack do not unfold simultaneously and their unfolded strands do not entangle with each other. Thus we observe the unfolding behavior of just the molecule on top.

**Fig 4:**
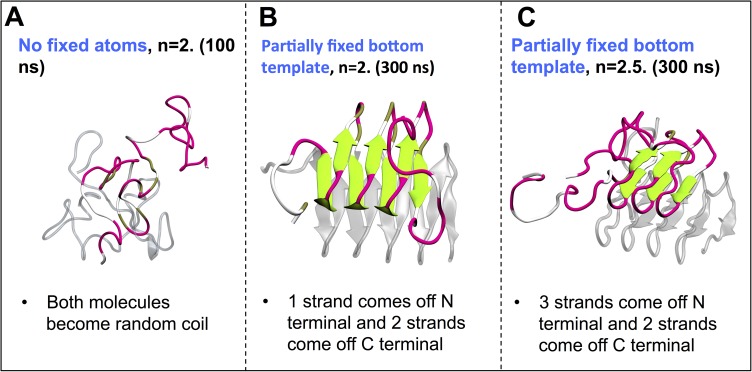
**The final structures from three different sets of simulations of systems with** (A) a two-molecule stack of β roll structures without fixed atoms at lowest threshold, (B) a two-molecule stack of β roll molecules with partially-fixed bottom template and intermediate threshold, and (C) a two-molecules stack of β roll molecules with partially-fixed bottom template and highest threshold. The bottom template is colored gray and is transparent. Random coil structures are in white, β turns are in pink and β sheets are in green.

Fig 4 shows the final structures in the three different types of simulations that we ran. The first type of simulation uses the threshold with *n* = 2 in Eq 7 and does not have any atoms fixed. As a result, both molecules in the stack in simulation 1 unfold completely after 80 ns; the snapshot in Fig 4 (A) is taken at the point at which Ω = 0 in Fig 3. A similar completely unfolded state occurs after 140 ns for simulation 2 in Fig 3. The second type of simulation is performed on a system that contains a partially-fixed bottom template and uses an intermediate threshold with *n* = 2 in Eq 7. As shown in Fig 4(B), the final structure of the top molecule in the stack has three unfolded strands: one off at the N terminus, and two off at the C terminus. The third type of simulation is again for a system with partially-fixed bottom template, and uses the highest threshold with *n* = 2.5. Now, five strands unfold from the top β roll molecule, as seen in Fig 4(C). This molecule does not unfold completely even after 300 ns of aMD simulations with an increased threshold, in stark contrast to the behavior observed in the system without partially-fixed template, where the chain collapses quickly when there are only 5 strands left in the β roll. These observations once again underline the importance of having a partially-fixed bottom template to stabilize the top β roll structure.

### Unfolding of a two-molecule stack with partially-fixed bottom template reproduces that of a stack without partially-fixed bottom template

**Fig 5:**
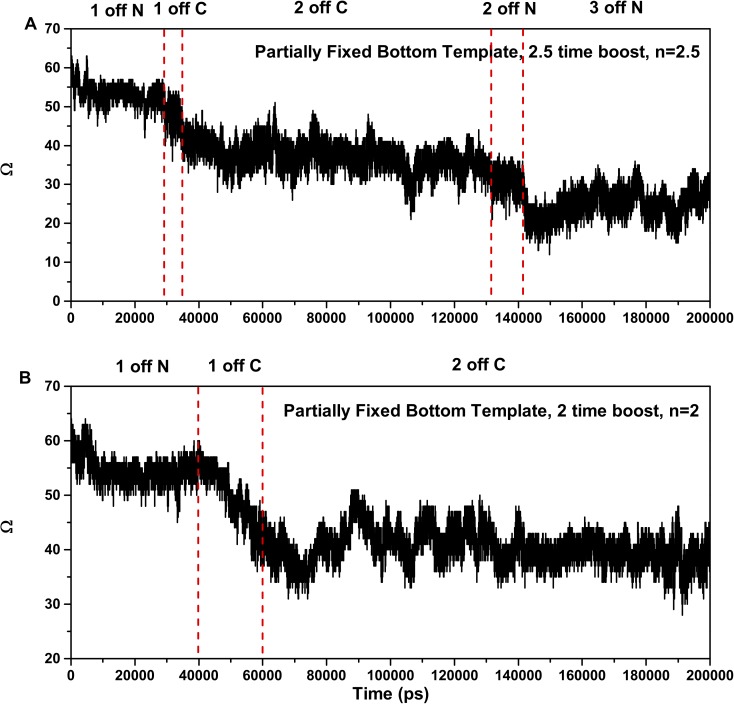
**Order parameter of the top molecule plotted against simulation time in simulations with partially-fixed bottom template and boosts (A) n = 2.5, and (B) n = 2.** The order parameter of both molecules are divided into several stages. Each stage corresponds to the unfolding stage labeled at the top of the graph.

The unfolding process for the top molecule in the two molecule stack with partially-fixed bottom template and boosts n = 2.0 or n = 2.5 resembles that of the molecule in the stack with no atoms fixed. Fig 5 plots the order parameters of the top molecule in the simulations with boost n = 2.5 and n = 2 against simulation time. The top molecule in the simulation with boost n = 2.5 (Fig 5A) shows that it goes through 5 stages to reach the final configuration, which has 3 strands coming off the N terminal and 2 strands coming off the C terminal. This stepwise unfolding is similar to the unfolding behavior of the top molecule in the stack without fixed atoms and n = 2 shown in Fig 2. The sequence of steps is: one strand comes off the N terminal, one strand comes off the C terminal, the second strand comes off the C terminal, the second strand comes off the N terminal, and finally the third strand comes off the N terminal. The only difference between the unfolding process for n = 2.5 with fixed atoms and n = 2 without fixed atoms is that the strands from N terminal come off earlier when n = 2.5 than when n = 2. The top molecule in the simulation with n = 2 and partially-fixed bottom template also unfolds in a step-wise fashion as shown in Fig 5B. Therefore, the unfolding behavior seems to be independent of the value of the boost potential.

### Lateral hydrogen bonding is stronger than vertical hydrogen bonding

**Fig 6:**
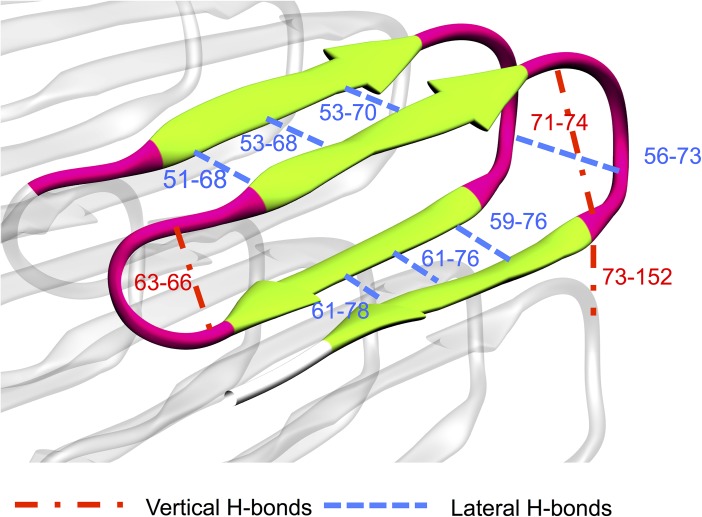
Illustration of lateral H-bonds and vertical H-bonds in the stack of two β roll molecules showing β strands (green) and β turns (pink) involved in the H-bonding calculation. Lateral H-bonds (blue dashed line) are between β sheets and between β turns. Vertical H-bonds (red dash-dot line) are within β turns or between a turn in the top molecule and a turn in the bottom molecule.

**Fig 7:**
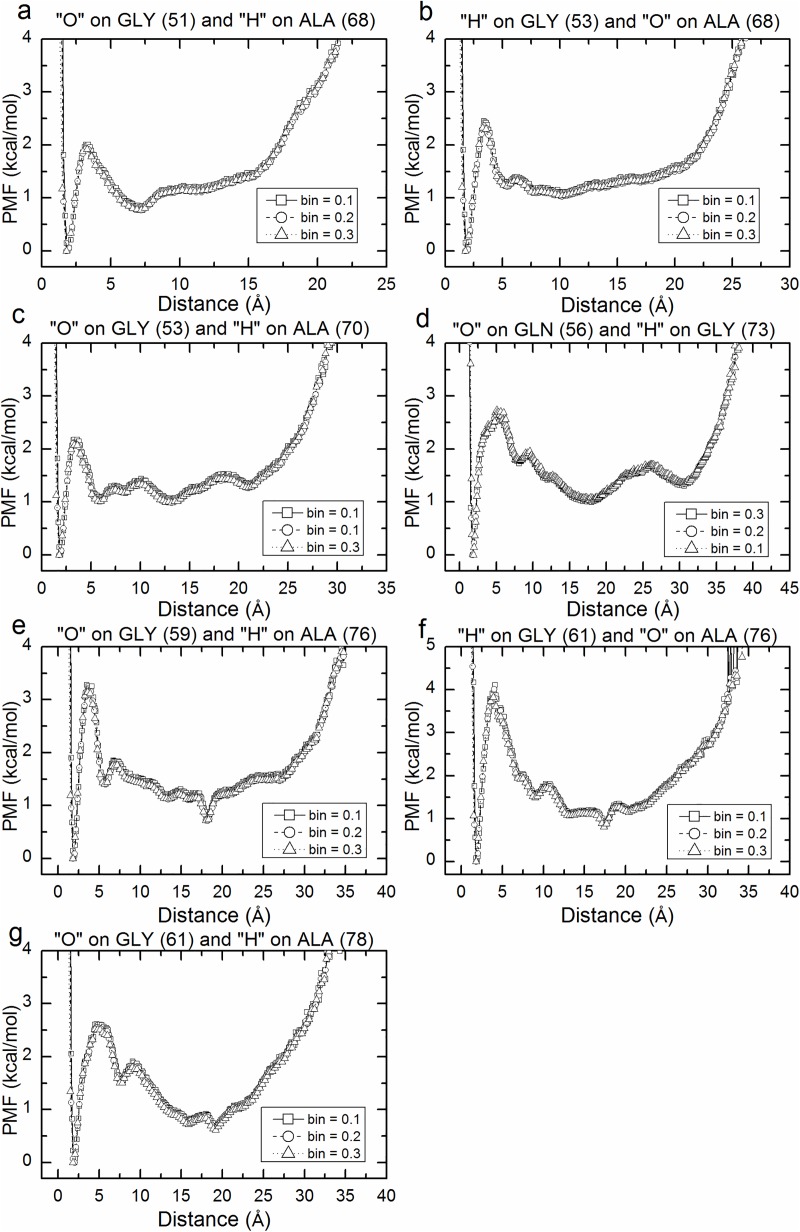
Unweighted PMF plotted against the distance between the lateral hydrogen bonded atom pairs shown in Fig 4 (blue dashed line) from one aMD simulation using boost potential n = 2 and partially-fixed bottom template. Three different bin sizes are shown in these plots. The hydrogen bonding pairs being presented are the oxygen atom on the 51th GLY residue and the hydrogen atom on the 68th ALA residue (a); the hydrogen atom on the 53rd GLY residue and oxygen atom on the 68th ALA residue (b); the oxygen atom on the 53th GLY residue and hydrogen atom on the 70th ALA residue (c); the oxygen atom on the 56th GLN residue and hydrogen atom on the 73rd GLY residue (d); the oxygen atom on the 59th GLY residue and hydrogen atom on the 76th ALA residue (e); the hydrogen atom on the 61st GLY residue and oxygen atom on the 76th ALA residue (f); and the oxygen atom on the 61st GLY residue and hydrogen atom on the 78th ALA residue (g).

**Fig 8:**
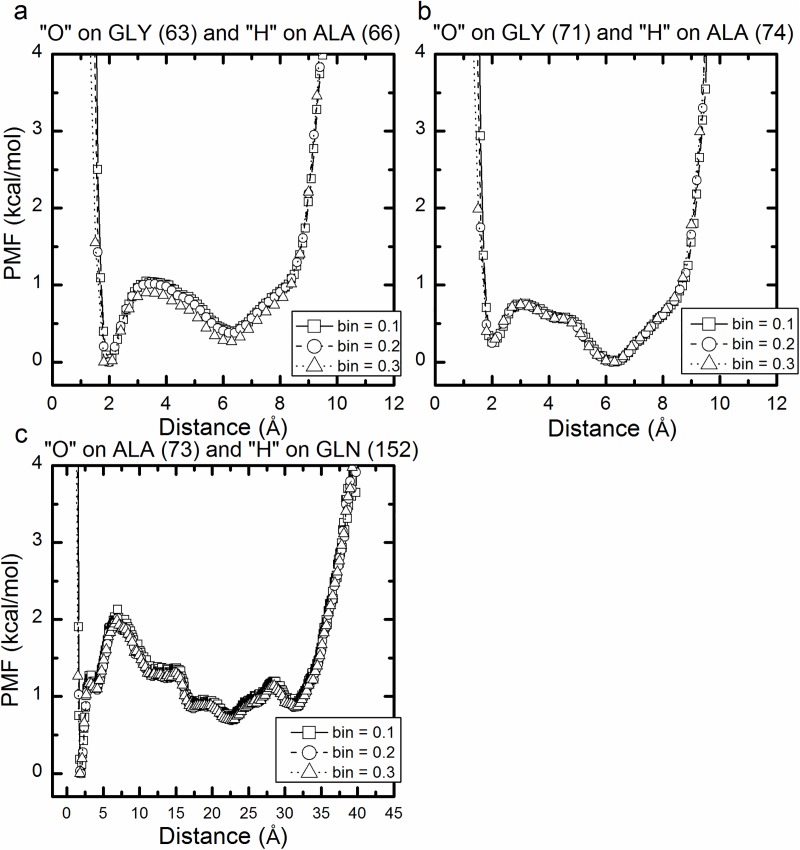
Unweighted PMF versus the distance between the vertical hydrogen bonded atoms pairs shown in Fig 4 (red dash-dot line) for one aMD simulation using boost potential n = 2 and partially-fixed bottom template. The hydrogen bonding pairs being presented are the oxygen atom on the 63rd GLY residue and the hydrogen atom on the 66th ALA residue (a); the oxygen atom on the 71st GLY residue and the hydrogen atom on the 74th ALA residue (b); the oxygen atom on the 73rd ALA residue and the hydrogen atom on the 152nd residue (c).

To identify the dominant forces in the β roll structure, we plot, for the selected hydrogen bonding atom pairs indicated in Fig 6, hydrogen bond potentials of mean force (PMF) versus distance in Figs 7 and 8. These are based on the trajectories generated by the aMD simulations with boost potential n = 2 and partially-fixed bottom template. Note that here we present the unweighted PMF versus the distance of hydrogen bonded pairs of only one of the two simulations-performed using boost potential n = 2 and partially fixed bottom template (recall that we performed two simulations for each set of parameters as shown in Table 1 in the method section). The unweighted PMF versus the distance of hydrogen bonded pairs of the other simulation is provided in the supporting information in S2 Fig and S3 Fig. Hydrogen bonds in a β roll configuration are categorized as being either lateral or vertical (see Fig 6). Lateral hydrogen bonds refer to the ones formed between the neighboring β strands in a single β sheet, or between neighboring β turns, and vertical hydrogen bonds refer to the ones between atoms in the top and bottom of a single β turn, or between atoms in the β turns of top and bottom molecules. All the unweighted potential of mean force profiles in Fig 7 (lateral H bonds) and 8 (vertical H bonds) are calculated with three different bin sizes, resulting in three curves for each plot. These curves match well with each other, indicating that we have enough samples for the calculation.

**Table 1:** Simulation lengths and threshold energies for the three different types of accelerated MD simulations (aMD).

Systems (2 simulations for each parameter)	Simulation Length (ns)	Thresholds used in aMD characterized by n^a^
Two-molecule β roll stack	100	Threshold with n = 2
Two-molecule β roll stack	200	Threshold with n = 2
Two-molecule β roll stack with partially-fixed bottom template	300	Threshold with n = 2
Two-molecule β roll stack with partially-fixed bottom template	400	Threshold with n = 2
Two-molecule β roll stack with partially-fixed bottom template	300	Threshold with n = 2.5
Two-molecule β roll stack with partially-fixed bottom template	400	Threshold with n = 2.5

^a^The number of acceleration factors, α_total,_ included in the threshold in Eq 3.

The unweighted PMFs associated with the hydrogen bonded atom pairs in Figs 7 and 8 have global minima at ~ 1.9 angstroms, indicating that these atoms prefer to stay within the hydrogen bonding distance. This reveals that the original β roll structure, in which all these atoms can form hydrogen bonds, is more stable than the unfolded structure, where only a few H bonds are possible. The hydrogen bonding strengths are taken to be the values of the PMF at the first peak in the PMF versus distance curves.

**Fig 9:**
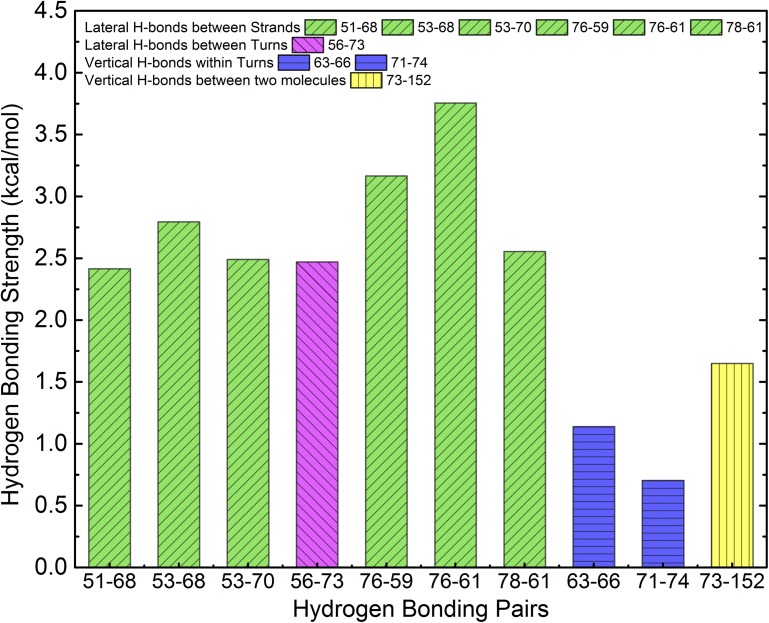
Hydrogen bonding strengths obtained from unweighted PMF profiles versus different hydrogen bonded residue pairs averaged over two aMD simulations with boost potential n = 2 and partially-fixed bottom template. Lateral H-bonds between strands (green) and lateral H-bonds between turns (pink), vertical H-bonds within turns (blue) and vertical H-bond between the two molecules (yellow).

The average hydrogen bond strengths in the two simulations with boost potential n = 2 and partially-fixed bottom template are given in Fig 9. The figure shows the hydrogen bonding strengths for the lateral hydrogen bonds between β strands (green) and between β turns (pink), and the vertical hydrogen bonds within β turns (blue) and between the turns in the top and bottom molecules (yellow). The first 3 columns represent the strengths of the lateral hydrogen bonds between the neighboring β strands in the top layer of the β roll structure as shown in Fig 6. The strengths of the lateral hydrogen bonds along the β strands in both the top and bottom layers of the β roll molecule are weaker when the hydrogen bonds are closer to the β turns: e.g. the hydrogen bonds between residues 51–68 and between residues 53–70 have a lower strength than the hydrogen bonds between residues 53–68. The strength of the lateral hydrogen bond formed between residues 56–73, indicated by the height of the 4th bar, is weaker than the lateral hydrogen bonds within the bottom layer but stronger than the lateral hydrogen bonds within the top layer of the top β roll molecule. Something similar is observed for the hydrogen bonds between neighboring β strands in the bottom layer of the β roll structure; see the 5th, 6th and 7th columns in Fig 9.

The lateral hydrogen bonds in the lower layer of the β roll structure are clearly stronger than those in the upper layer of the β roll structure. As seen in Fig 9, the 5th, 6th and 7th columns representing the hydrogen bonding strength in the bottom layer of the β roll, are higher than the first three columns representing the hydrogen bonding strength in the top layer of the β roll. This is likely a consequence of the bottom layer in the top molecule being in direct contact with the bottom template. The effect of stacking on the stability of the roll was investigated in our previous study [7]; there we found that stacking helps stabilize the β roll structure by increasing the number of intra-molecular hydrogen bonds in each β roll molecule. Here we see that in terms of bond energies that conclusion is confirmed.

The vertical hydrogen bonds are usually weaker than the lateral hydrogen bonds. This can be observed by comparing the heights in Fig 9 of the first 7 columns, which represent the strengths of the lateral hydrogen bonds, with the heights of the last 3 columns, which represent the strengths of the vertical hydrogen bonds. The heights of the two blue columns in Fig 9, which represent hydrogen bonds within the β turns, are smaller than those of the first 7 columns, representing the lateral hydrogen bonds. This signifies that lateral hydrogen bonds play a more important roll than vertical hydrogen bonds in keeping the molecule in a β roll configuration. The height of the yellow bar, which represents the average strength of the hydrogen bond between the top and bottom molecules (there is one such bond per strand), is slightly lower than that of the first three columns, indicating that the hydrogen bonds between the two molecules are almost as strong as the lateral hydrogen bonds between the β strands in the upper layer of the top molecule. This suggests that the hydrogen bonds between the two molecules also play a significant role in maintaining the β roll structure of the top molecule.

### Higher threshold simulations reveal other potential structures and self-healing ability

**Fig 10:**
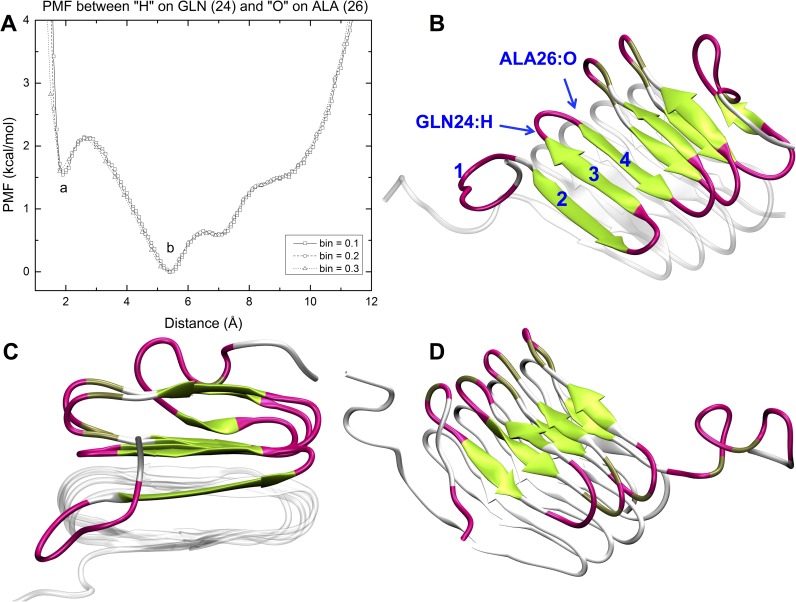
The PMF profile of hydrogen atom on GLN and the oxygen atom on ALA and corresponding representative structure at each minimum. The unweighted potential mean force associated with hydrogen atom on GLN and the oxygen atom on ALA is plotted against the distance between the two atoms (A). Two minima are identified, a local minimum, a, and a global minimum, b. An intermediate structure (B) and its side view (C) correspond to the energy local minimum a. A distorted β roll structure associated with the global minimum of energy, b, is shown as (D).

In the simulation with the highest threshold energy, where n = 2.5 in Eq 7, and a partially-fixed bottom template, a new intermediate structure shows up. It contains a β hairpin structure and an anti-parallel β sheet formed by strands from the top and bottom molecules. Fig 10(A) shows the unweighted PMF versus the distance between the hydrogen on GLN (residue 24) and the oxygen on ALA (residue 26). Two minima are identified in the plot, a local minimum at short distance (a) and a global minimum further out (b). The intermediate structure associated with the local minimum is shown in Fig 10(B) and its side view is shown in Fig 10(C). The structure associated with the global minimum is a distorted β roll structure as shown in Fig 10(D). The potential well of the intermediate structure is located at ~ 2 angstroms, indicating that a hydrogen bond forms between the hydrogen on GLN (residue 24) and the oxygen on ALA (residue 26), i.e., residues 24, 25 and 26 have formed a three-amino-acid turn. Strands 3 and 4 form an antiparallel β sheet structure. Taken together, the turn and the antiparallel structures are essentially a typical β hairpin structure. Another anti-parallel β sheet is formed between strand 2 in the top molecule and the silver strand in the bottom molecule. A side view of this structure is seen in Fig 10(C) which shows how strands 2 and 3 traverse the interface between the two molecules. The reason this structure forms is that the first strand from the N terminal in the bottom template is not fixed and breaks loose. This provides enough room for the second and third strands of the top molecule to reach down one layer, forming β sheets with the strand in the bottom template.

The anti-parallel β sheet formed by strands from the top and bottom molecules in the intermediate structure is of particular interest to us as this configuration could potentially inhibit the unfolding process. In a long fibril with many molecules, the molecules in a β roll structure will probably not always stack as perfectly as in our starting configuration, meaning that strands from some molecules could potentially form β sheets with their folded neighbors, thus preventing the unfolding process. We call this a self-healing ability because it seems to hinder the β roll molecule from completely unwrapping; it might be one reason why the fibrils observed in the experiments are very strong.

Considering the results obtained here with respect to unfolding, we tentatively propose a hypothetical folding pathway; we emphasize that this is highly speculative and should not be considered as a conclusion supported by the simulation data obtained here, but rather as a direction for further studies. The folding process most likely starts with docking of a disordered silk-like (GAGAGAGX)_n_ domain on a pre-folded molecule acting as template. This consistent with the observation that the template provides stability to the folded roll, and with the experimental fact that secondary structure develops in parallel with fibril growth. The disordered domain has to remain long enough in the docked state to allow for nucleation of a minimal folded part, *e*.*g*., a 5-stranded β solenoid. This step has a very low probability and is therefore likely to be rate-determining, accounting for the very low growth rates observed experimentally^11^. Moreover, it also would explain why silk-like domains of higher number of repeating units, which are likely to have longer residence times and a higher nucleation probability, tend to give faster growth [23]. Once nucleation has occurred, the remainder of the silk-like domain can fold to form the complete β solenoid.

## Discussion

We used accelerated molecular dynamics (aMD) simulations to investigate the unfolding of a stack of two β roll molecules, (GAGAGAGQ)_10_. Although much is known of about the structure of the β solenoid, very little is known about the partially folded conformation of the silk-like polypeptide or the details of the folding/unfolding process. Unfolding simulations can help us understand biological processes and, when well sampled, can provide us with partially-folded structures. aMD is able to maintain the original shape of the energy landscape and let the molecule sample conformational space fairly naturally.

Our goal was to identify the dominant forces that keep the silk-inspired polypeptide in a β roll configuration, to investigate the unfolding mechanism of silk-inspired polypeptides. The β roll structure that we use in this study was obtained from our previous investigation of the stable configuration of the β roll using the same sequence (GAGAGAGA)_10_. Unlike the structure proposed by Schor et al. [14] where all the alanine residues pointed out, forming a hydrophobic shell, the structure used in this study possesses a hydrophobic core which we have shown to be more stable than that with a hydrophobic shell [7].

The size of the boost potential was chosen carefully. It should not be so small that unfolding is unlikely to occur, as this would essentially be the same as a conventional MD simulation and it should not be too strong, because this might induce an unrealistic unfolding process. The number of boost potentials added to the original potential, n, was therefore chosen to reveal both the unfolding and the any spontaneous refolding of the polypeptide that occurred during the simulations. The unfolding process of the molecule on top without any fixed atoms showed rejoining of the strands as well as unfolding. Moreover, we saw that the unfolding process can be reproduced by additional simulations with the same parameters and even by simulations with different boost potentials.

To justify the convergence of our simulations, the relaxation time of the peptide backbone vectors is estimated from the time autocorrelation function profile. S1 Fig show a plot of the time correlation function of the out-of-plane vectors (the vector that is perpendicular to the plane formed by 3 consecutive carbon atoms) of the polypeptide versus the simulation time. The time at which this reaches zero gives a measure of the relaxation time of the peptide [7]. The relaxation time is less than 10 ns for simulations without fixed atoms, indicating that our 100ns simulation is long enough to reach equilibrium. The 300 ns simulations with partially-fixed bottom template have relaxation times less than 100 ns, which indicates that the molecules in these systems have reached equilibrium.

By comparing the unfolding order parameter, Ω, versus simulation time between cMD and aMD, we found that a molecule in a stack of two β roll molecules unfolds in a step-wise fashion, i.e. one β strand in the β roll molecule at a time, which agrees well with the experimental study on transmembrane β-barrel protein OmpG by Sapra et al. [21]. We also found that it unfolds mainly from the C terminal, which matches with the simulation study on a prototypic TpsA protein, FHA by Alsteens et. al [22]. Through observing the unfolding and spontaneous refolding of single strand in the β roll structure, we get a better idea of the possible intermediates that might occur during the folding process. Schor et al. [15] hypothesize that the molecule folds into a β roll structure with a hydrophobic shell by itself, then docks onto another preformed β roll molecule, a “roll n’ dock” process.

The bottom template is found to play an important role in stabilizing the β roll structure of the molecule on top. This was concluded by comparing the final structure in three sets of simulations with systematically increased threshold energies. At the lowest threshold energy, both molecules unfold and have a random coil structure by the end of the simulation for systems without any fixed atoms. When the bottom template is partially fixed, the top molecule is unable to unfold completely, even by the end of 300 ns simulations, indicating the significance of the bottom template in stabilizing the molecule on top of it.

We further elucidate how the bottom template stabilizes the top β role molecule by quantifying the strengths of the various intra and inter molecular hydrogen bonds. The lateral hydrogen bonds in the lower layer of the top molecule are stronger than those in its upper layer, indicating that the bottom template strengthens the hydrogen bonds in the lower layer of the top molecule. We further confirm the stabilizing effect of the bottom template reported in our previous investigation, in which we concluded that the bottom template induces more intramolecular hydrogen bonds in the top molecule when it docks on to the bottom template[7]. We also found that the lateral hydrogen bonds between the β strands in a β roll configuration become weaker as they get close to the β turns. This is due to the fact that the β turn structure is more flexible than the β sheet structure in the β roll molecule. Vertical hydrogen bonds within the β roll structure are considerably weaker than lateral hydrogen bonds, signifying the importance of lateral hydrogen bonds in stabilizing the β roll structure.

Finally, an intermediate structure was found containing a β hairpin and an anti-parallel β sheet formed by strands from the top and bottom molecules, revealing the self-healing ability of the β roll stack. The β hairpin structures can form fibrils by themselves as reported by other studies [22, 23] in which β hairpins first stack by hydrophobic interactions and then assemble via hydrogen bonds. Here we found β hairpins in the stack of two silk-inspired molecules, indicating that these β hairpins may also play a role in stabilizing silk-inspired fibrils with many molecules. Such β sheets formed between pairs of molecules, an inter-protein β sheet structure, were also reported by Razzokov et al., who studied a sequence similar to ours, (GAGAGAGE)_5_, using replica exchange molecular dynamics[24]. Overall, the strength of the fibril of β roll molecules comes not only from the stability of each individual molecule, but also from the cooperative effect provided by the anti-parallel β sheet structures formed by strands from the top and bottom molecules. Our results led us to tentatively propose a hypothetical folding pathway that is consistent with the experimental results.

## Methods

### Conventional molecular dynamics

In our previous paper^7^, we performed conventional explicit-solvent atomistic molecular dynamics simulations on a stack of two β roll molecules with a sequence (GAGAGAGQ)_10_ using Amber 12 and the ff12SB force field. The simulation details can be found in our previous paper[7). The last 50 ns of those trajectories were used to calculate the time averages of the total potential energy of the system as well as the dihedral energy of the peptides.

### General form of accelerated molecular dynamic

**Fig 11:**
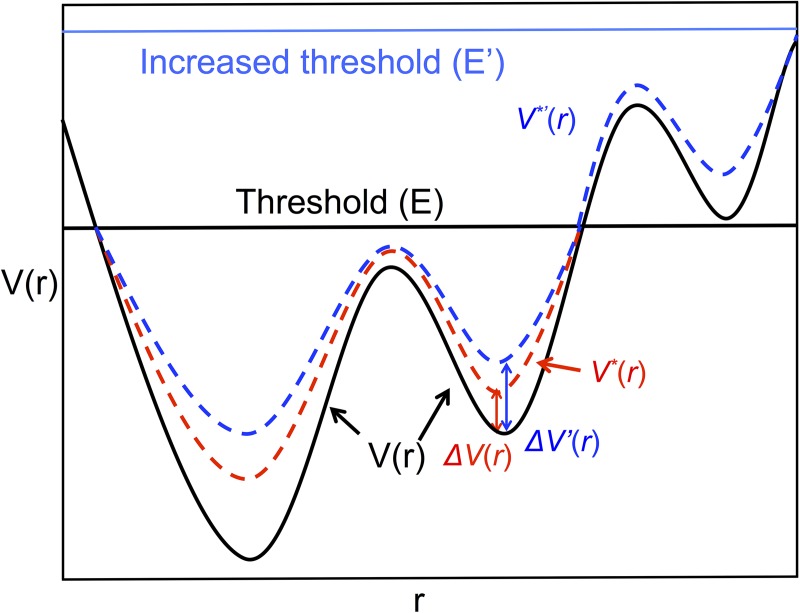
Illustration of aMD simulation. Graph of V(r) versus r showing a low threshold potential energy, E, (black horizontal line) and a high threshold potential energy, E’, (blue horizontal line). In an aMD simulation, a boost ΔV(r) is added to the original potential energy landscape V(r) when the system potential or the dihedral energy is lower than the predefined threshold E, resulting in the boosted potential energy, V*(r) (dashed red line). Regions of the potential energy curve that are above the threshold energy maintain their original form. If the threshold energy is increased to E’, a larger boost potential energy, ΔV’(r) is added to the original free energy. In that case another free energy minimum goes below the new threshold, E’, resulting in a boost potential added to that region. The shape of the energy surface and the position of the energy minimum remain the same after adding the boost.

The general principles of aMD are as follows. A boost potential Δ*V*(*r*) is added to the original potential energy surface of the system when the system’s potential energy is lower than a predefined threshold energy, *E* [25], as shown in Fig 11.

$$\begin{array}{l} V*(r)=V(r)+\Delta V(r),\;V(r)<E,\\ V*(r)=V(r),\;V(r)\geq E, \end{array}$$ (1)

where V*(r) is the modified (boosted) potential energy, V(r) is the original potential energy (which could be the total potential energy of the system or the dihedral energy of the polypeptide) and r is a positional degree of freedom or a torsional degree of freedom, etc. The general form of the boost potential, Δ*V*(*r*) is given by the equation below:

$$\Delta V(r)=\frac{{\left(E-V(r)\right)}^{2}}{\alpha +E-V(r)}$$ (2)

where *α* is the acceleration factor. The acceleration factor is a parameter that governs the size of the boost. As it gets smaller, the energy surface becomes flatter, thus improving the likelihood of transitions between low energy states. The gist of the method therefore is that the global pattern of the potential energy is maintained, but barriers become smaller, allowing easier passage.

### Dual-boost accelerated molecular dynamics

Dual-boost aMD simulations are performed in our investigation, which means boost potential energies are added to both the total potential energy of the system and to the dihedral energy of the polypeptides. The total potential energy of the system V_total_ (r) consists of the dihedral energy V_dihedral_(r) and the non-dihedral energy V_non-dihedral_(r) as shown below,

$$V_{total}(r)=V_{non-dihedral}(r)+V_{dihedral}(r).$$ (3)

In a dual boost aMD simulation, a boost potential ΔV_dihedral_(r) is first added to the dihedral energy of the peptide as in Eq 4 below,

$$V_{dihedral}^{*}(r)=V_{dihedral}(r)+\Delta V_{dihedral}(r)$$ (4)

where $V_{dihedral}^{*}(r)$ is the modified dihedral energy of the peptide. Then a boost potential ΔV_total_(r) is added to the total potential energy of the system as shown in Eq 5 below,

$$V_{total}^{*}(r)=\{V_{non-dihedral}(r)+V_{dihedral}^{*}(r)\}+\Delta V_{total}(r)$$ (5)

where $V_{total}^{*}(r)$ is the modified total potential energy and ΔV_total_(r) is the boost potential energy added to the total potential energy of the system, V_total_(r).

The equations used to calculate the boost potential of the total potential energy of the system V_total_ (r) and the boost potential of the dihedral energy V_dihedral_(r) are

$$\begin{array}{l} \Delta V_{total}(r)=\frac{{\left(E_{total}-V_{total}(r)\right)}^{2}}{\alpha_{total}+E_{total}-V_{total}(r)}\\ \Delta V_{dihedral}(r)=\frac{{\left(E_{dihedral}-V_{dihedral}(r)\right)}^{2}}{\alpha_{dihedral}+E_{dihedral}-V_{dihedral}(r)} \end{array}$$ (6)

where *E*_total_ and *E*_dihedral_ are the thresholds for the total energy and dihedral energy, and *α*_total_ and *α*_dihedral_ are the acceleration factors for the total potential energy and dihedral energy. Note that these equations are of the same form as Eq 2.

The pre-defined thresholds, *E*_total_ and *E*_dihedral_, and the acceleration factors, *α*_total_ and *α*_dihedral_, for the two types of boosts are calculated as below,

$$\begin{array}{l} \;E_{dihedral}=V_{avg\_dihedral}+3.5\times N_{res},\;\alpha_{dihedral}=3.5\times N_{res}/5\\ E_{total}=V_{avg\_total}+n\times \alpha_{total},\;\alpha_{total}=0.2\times N_{atoms}\;\left(n=1,2,3\ldots \right) \end{array}$$ (7)

where *V*_avg_dihed_ and *V*_avg_total_ are time averages of the dihedral energy and total potential energy obtained from conventional molecular dynamics (cMD). These are calculated only once, before running the aMD simulations, to generate the value of thresholds. The parameters *N*_res_ and *N*_atoms_ are the number of polypeptide residues and the number of atoms in the system, respectively; *n* in Eq 7 is an integer that determines the magnitude of the threshold as a multiple of the acceleration factor.

Consequently, there are only four input parameters in an aMD simulation: *E*_dihedral_, α_dihedral_, E_total_ and α_total_. All the other parameters are calculated based on these four values. Sometimes we increase the threshold of the total potential, *E*_total_, by making the value of *n* in Eq 7 larger, to let the system access more conformational space. Increasing the threshold of the total potential energy enables more energy minima to lie below the threshold and have boost potentials added to them. As shown in Fig 11, the third free energy minimum from the left does not get an added boost potential when the threshold is E. When the threshold to is increased to E’, the third minimum falls below the threshold, so a boost potential is added to it. This facilitates the transition between the second and the third minima. We used different threshold energies in different sets of simulations in our investigation, so the value of *n* in Eq 7 varies from simulation to simulation.

### aMD simulation of the β roll stack

**Fig 12:**
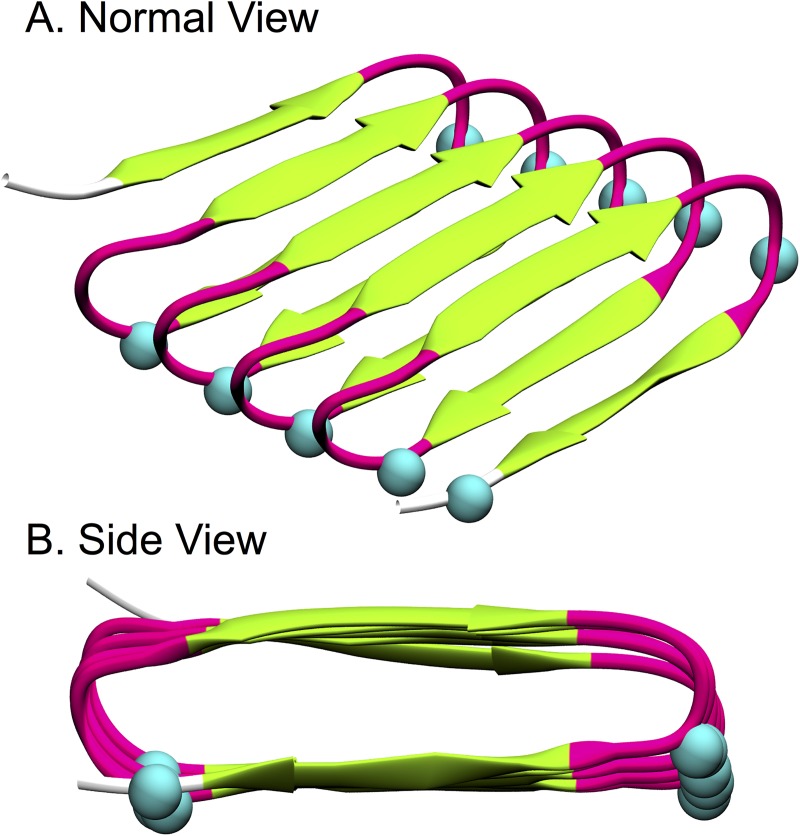
**Illustration of fixed atoms in the bottom β roll molecule with sequence (GAGAGAGQ)**_**10**_ **in both normal view (A) and side view (B).** The fixed atoms (cyan beads) are the Cα atoms on the glycine residues on each side of the β roll below the β turns (in pink). The parallel β sheets are in green and random structures are in white.

We ran three types of aMD simulations on the stack of two **β** roll molecules (GAGAGAGQ)12_10_ shown in Fig 1 and two simulations for each set of aMD boost parameter as shown in Table 1. The first type of simulation uses the lowest threshold potential energy with n = 2 in Eq 7. This type of simulation is performed for 100 ns. The second and the third type of simulations use increased threshold potential energies with n = 2 and 2.5, respectively, and are performed for 300 ns each. During the first type of simulation, no atoms are fixed. However, during the second and the third types of simulations, 10 C_α_ atoms in the glycine (G) residues at the bottom of the β turns on both sides of the β roll molecule are spatially fixed to maintain the bottom template’s β roll configuration, as shown in Fig 12. These atoms are restrained with a force of 10 kcal/mol, and were chosen to mimic the presence of a substrate. In experiment, a substrate is used to grow fibrils; the fibrils are found to grow perpendicular to the substrate [16,26]. Fixing specific atoms in the bottom β roll molecule helps maintain the bottom molecule in a β roll configuration, while allowing some flexibility to the chain. The simulation times, as well as the number of acceleration factors added to the average system’s total potential energy for different types of simulations are summarized in Table 1.

### Potential mean force calculation

Hydrogen bonds play a very important role in keeping the folded and stacked structure together. We therefore pay specific attention to the strength of these bonds, by calculating potential of mean force (PMF) profiles. Potential mean force (PMF), used synonymously in the literature to indicate a free energy profile, examines the change of a system’s free energy as a function of some specific reaction coordinate, such as the lateral distance between the hydrogen bonding sites on two neighboring β strands or the vertical distance between hydrogen bonding sites within a **β** turn structure or between the two stacking molecules. In this work we take as our reaction coordinates the distances between the important hydrogen bonded atoms pairs. The strength of the hydrogen bonds can be gleaned from the height of the first peak in these PMF profiles. The dominant forces controlling docking and folding of the **β** roll can then be determined by comparing the strengths of these hydrogen bonds. Usually, the free energy profiles generated from the aMD simulations need to be reweighted using the Boltzmann factor for the boost potential [17]. However, given the large size of our system, there is a large noise associated with reweighting the free energy profile. Therefore, and since we use the profiles only for comparative purposes, unweighted PMF profiles are presented in this paper. A similar approach was used in a study of the free energy landscape of large systems of G-protein coupled receptors [19] by Miao et al.

The unweighted potential of mean force or F(A_j_) is calculated as a function of the distance A_j_ between hydrogen-bonded partners, for different atoms pairs. The relevant equation is F(A_j_) = -k_B_Tln p(A_j_), where p (A_j_) signifies the probability of finding the atoms pairs within A_j_ (distances are divided into a number of equally distributed bins with index j), k_B_ is the Boltzmann constant, and T is the temperature of the system.

The hydrogen bonded atom pairs used to calculate the potential mean force (PMF) are constantly forming and breaking with time. S1 Table gives the hydrogen-bonding average lifetimes which were calculated in the following way. In the hydrogen-bonding data set, we define a hydrogen bonding indicator for each pair of atoms to be 1.0 when they are hydrogen bonded and 0.0 when they are not. The average lifetime is determined by averaging the length of time that a particular hydrogen bond is present continuously. As shown in S1 Table, the number of times that the hydrogen bonds form and break varies from 2007 times to 6808 time. The maximum lifetime for hydrogen bonded atom pairs in PMF calculations is between 50 ps and 223 ps. The average lifetime for atom pairs in PMF calculations is from 6.123 ps to 24.0842 ps. It is apparent that the hydrogen bonds are constantly breaking and forming, over our simulations which of to 300,000 ps.

### Unfolding order parameter

In order to characterize the state of molecules with respect to folding, an unfolding order parameter, Ω, is needed which measures departures of the β roll from its ideal structure. We here define Ω as the number of amino acids that are at an appropriate distance to the neighbor they would have in an ideal β roll structure, *e*.*g*., by counting pairs of residues i and i+16, that are within a distance of 4.0 to 6.0 angstroms of each other. This typical range has been inferred from cMD simulations during which the molecules retain their β roll structure. When the (GAGAGAGQ)_10_ peptide (which has a total of 80 residues) is in a perfect β roll structure there are 64 pairs (i, i + 16), so that the maximum value of Ω is 64.

## Supporting information

S1 Fig Time correlation function of peptide backbone Cα-Cα bond unit vectors out of plane. Relaxation time of peptide backbone vectors can be estimated from time autocorrelation profile for (A) peptides without fixed atoms and boost n = 2 (B) peptides with partially fixed bottom template and boost n = 2 and (C) peptides with partially fixed bottom template and boost n = 2.5.
(TIF)

S2 Fig Unweighted PMF plotted against the distance between the lateral hydrogen bonded atom pairs shown in Fig 4 (blue dashed line from aMD). This is for the second set of aMD simulations using boost potential n = 2 and partially-fixed bottom template.
(TIF)

S3 Fig Unweighted PMF plotted against the distance between the vertical hydrogen bonded atom pairs. This is for the second set of aMD simulations using boost potential n = 2 and partially-fixed bottom template.
(TIF)

S1 Table Hydrogen bonding lifetime for Lateral and Vertical Hydrogen-bonded Atom Pairs. (XLSX)

## References

[1] Lin F-H, Davies PL, Graham LA. The Thr- and Ala-Rich Hyperactive Antifreeze Protein from Inchworm Folds as a Flat Silk-like β-Helix. Biochemistry. 2011 5 31;50(21):4467–78. 10.1021/bi2003108 21486083

[2] Middleton AJ, Marshall CB, Faucher F, Bar-Dolev M, Braslavsky I, Campbell RL, et al Antifreeze Protein from Freeze-Tolerant Grass Has a Beta-Roll Fold with an Irregularly Structured Ice-Binding Site. Journal of Molecular Biology. Elsevier Ltd; 2012 3 9;416(5):713–24. 10.1016/j.jmb.2012.01.032 22306740

[3] Graether SP, Kuiper MJ, Gagne SM, Walker VK, Jia Z, Sykes BD, et al β-Helix structure and ice-binding properties of a hyperactive antifreeze protein from an insect. Nature. 2000;406(6793):325–8. 10.1038/35018610 10917537

[4] Barnhart MM, Chapman MR. Curli Biogenesis and Function. Annu Rev Microbiol. 2006 10;60(1):131–47. 16704339 10.1146/annurev.micro.60.080805.142106 PMC2838481

[5] Kisker C, Schindelin H, Rees DC. A left-handed β-helix revealed by the crystal structure of a carbonic anhydrase from an archaeon. Acta Crystallogr A Found Crystallogr. 1996 8;52(a1):C168–8. PMC450161 8665839

[6] Kajava AV, Steven AC. β‐Rolls, β‐Helices, and Other β‐Solenoid Proteins In: Fibrous Proteins: Amyloids, Prions and Beta Proteins. Elsevier; 2006 pp. 55–96. (Advances in Protein Chemistry; vol. 73). 10.1016/S0065-3233(06)73003-0 17190611

[7] Zhao B, Cohen Stuart MA, Hall CK. Dock 'n roll: folding of a silk-inspired polypeptide into an amyloid-like beta solenoid. Soft Matter. 2016 4 20;12(16):3721–9. 10.1039/c6sm00169f 26947809 PMC4913789

[8] Hernandez-Garcia A, Kraft DJ, Janssen AFJ, Bomans PHH, Sommerdijk NAJM, Thies-Weesie DME, et al Design and self-assembly of simple coat proteins for artificial viruses. Nature Nanotechnology. Nature Publishing Group; 2014 8 24;9(9):698–702. 10.1038/nnano.2014.169 25150720

[9] Yan Y, Martens AA, Besseling NAM, de Wolf FA, de Keizer A, Drechsler M, et al Nanoribbons Self-Assembled from Triblock Peptide Polymers and Coordination Polymers. Angewandte Chemie International Edition. 2008 5 19;47(22):4192–5. 10.1002/anie.200705242 18428166

[10] Martens AA, Portale G, Werten MWT, de Vries RJ, Eggink G, Cohen Stuart MA, et al Triblock Protein Copolymers Forming Supramolecular Nanotapes and pH-Responsive Gels. Macromolecules. 2009 2 24;42(4):1002–9.

[11] Włodarczyk-Biegun MK, Werten MWT, de Wolf FA, van den Beucken JJJP, Leeuwenburgh SCG, Kamperman M, et al Genetically engineered silk–collagen-like copolymer for biomedical applications: Production, characterization and evaluation of cellular response. Acta Biomater. 2014 8 1;10(8):3620–9. 10.1016/j.actbio.2014.05.006 24814883

[12] Peralta MDR, Karsai A, Ngo A, Sierra C, Fong KT, Hayre NR, et al Engineering Amyloid Fibrils from β-Solenoid Proteins for Biomaterials Applications. ACS Nano. 2015 1 27;9(1):449–63. 10.1021/nn5056089 25562726

[13] Beun LH, Beaudoux XJ, Kleijn JM, de Wolf FA, Stuart MAC. Self-assembly of silk-collagen-like triblock copolymers resembles a supramolecular living polymerization. ACS Nano. 2012 1 24;6(1):133–40. 10.1021/nn203092u 22168567

[14] Schor M, Martens AA, deWolf FA, Cohen Stuart MA, Bolhuis PG. Prediction of solvent dependent β-roll formation of a self-assembling silk-like protein domain. Soft Matter. 2009;5(13):2658–8.

[15] Schor M, Bolhuis PG. The self-assembly mechanism of fibril-forming silk-based block copolymers. Phys Chem Chem Phys. 2011;13(22):10457–11. 10.1039/c0cp02842h 21503301

[16] Ni R, Kleijn JM, Abeln S, Cohen Stuart MA, Bolhuis PG. Competition between surface adsorption and folding of fibril-forming polypeptides. Phys Rev E Stat Nonlin Soft Matter Phys. 2015 2;91(2):022711 10.1103/PhysRevE.91.022711 25768535

[17] Miao Y, Sinko W, Pierce L, Bucher D, Walker RC, McCammon JA. Improved Reweighting of Accelerated Molecular Dynamics Simulations for Free Energy Calculation. J Chem Theory Comput. 2014 7 8;10(7):2677–89. 10.1021/ct500090q 25061441 PMC4095935

[18] Pierce LCT, Salomon-Ferrer R, Augusto F de Oliveira C, McCammon JA, Walker RC. Routine Access to Millisecond Time Scale Events with Accelerated Molecular Dynamics. J Chem Theory Comput. 2012 9 11;8(9):2997–3002. 10.1021/ct300284c 22984356 PMC3438784

[19] Miao Y, Nichols SE, McCammon JA. Free energy landscape of G-protein coupled receptors, explored by accelerated molecular dynamics. Phys Chem Chem Phys. 2014;16(14):6398–9. 10.1039/c3cp53962h 24445284 PMC3960983

[20] Song J, Li Y, Ji C, Zhang JZH. Functional Loop Dynamics of the Streptavidin-Biotin Complex. Sci Rep. 2015 1 20;5:7906–10. 10.1038/srep07906 25601277 PMC4298722

[21] Sapra KT, Damaghi M, Köster S, Yildiz Ö, Kühlbrandt W, Muller DJ. One β Hairpin after the Other: Exploring Mechanical Unfolding Pathways of the Transmembrane β-Barrel Protein OmpG. Angewandte Chemie International Edition. 2009 9 28;48(44):8306–8. 10.1002/anie.200904361 19787673

[22] Alsteens D, Martinez N, Jamin M, Jacob-Dubuisson F. Sequential unfolding of beta helical protein by single-molecule atomic force microscopy. PLoS ONE. 2013;8(8):e73572 10.1371/journal.pone.0073572 24009757 PMC3756990

[23] Beun LH, Storm IM, Werten MWT, de Wolf FA, Cohen Stuart MA, de Vries R. From micelles to fibers: balancing self-assembling and random coiling domains in pH-responsive silk-collagen-like protein-based polymers. Biomacromolecules. 2014 9 8;15(9):3349–57. 10.1021/bm500826y 25133990 PMC4260859

[24] Razzokov J, Naderi S, van der Schoot P. Prediction of the structure of a silk-like protein in oligomeric states using explicit and implicit solvent models. Soft Matter. 2014;10(29):5362–13. 10.1039/c4sm00384e 24937549

[25] Hamelberg D, Mongan J, McCammon JA. Accelerated molecular dynamics: A promising and efficient simulation method for biomolecules. J Chem Phys. AIP Publishing; 2004 6 22;120(24):11919–29. 10.1063/1.1755656 15268227

[26] Charbonneau C, Kleijn JM, Cohen Stuart MA. Subtle charge balance controls surface-nucleated self-assembly of designed biopolymers. ACS Nano. 2014 3 25;8(3):2328–35. 10.1021/nn405799t 24571369

