# Supplementary figures and images for: Navigating in foldonia: Using accelerated molecular dynamics to explore stability, unfolding and self-healing of the β-solenoid structure formed by a silk-like polypeptide
Source: PLoS Comput Biol. 2017 Mar 22;13(3):e1005446. doi: 10.1371/journal.pcbi.1005446 (PMC5388506; doi:10.1371/journal.pcbi.1005446)

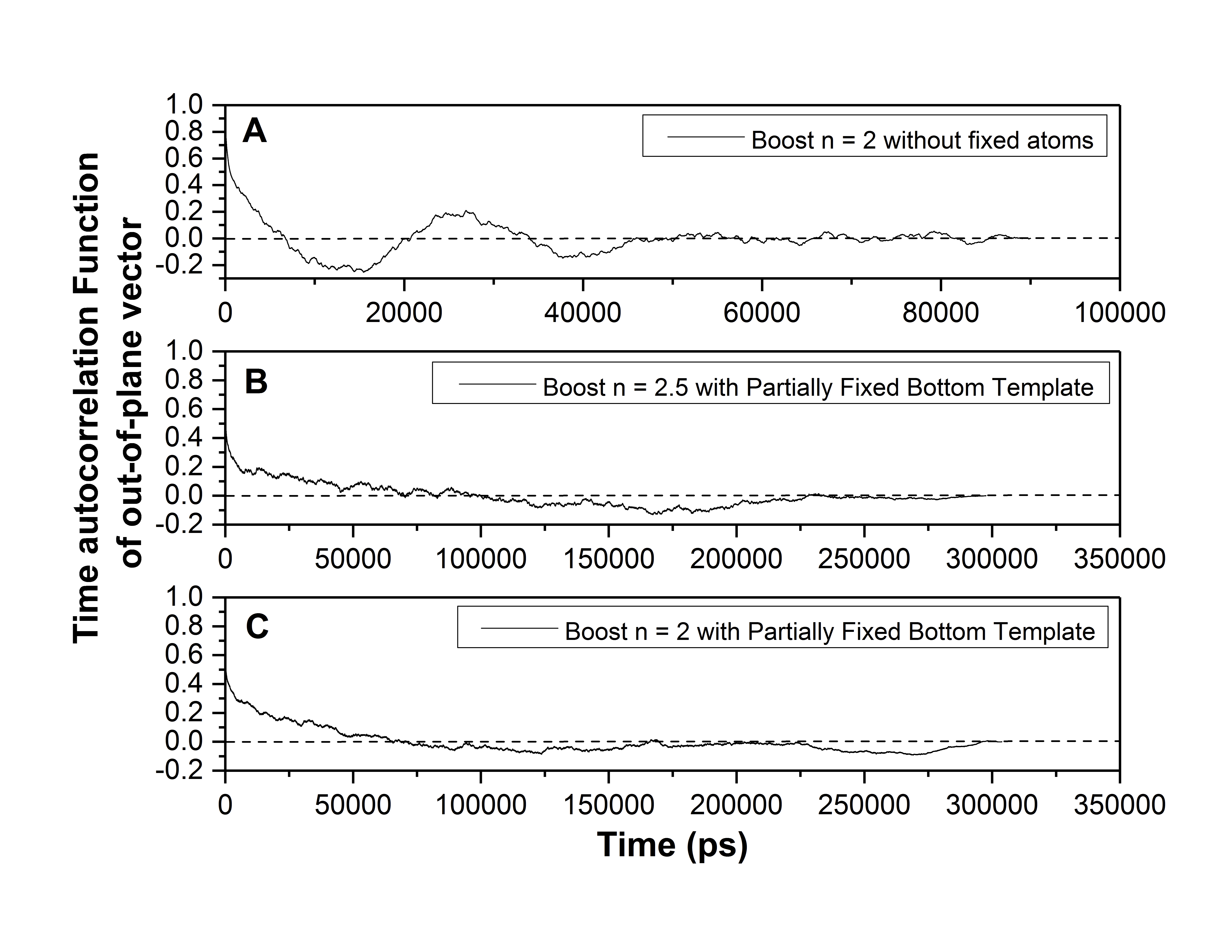

Supplement: S1 Fig — Relaxation time of peptide backbone vectors can be estimated from time autocorrelation profile for (A) peptides without fixed atoms and boost n = 2 (B) peptides with partially fixed bottom template and boost n = 2 and (C) peptides with partially fixed bottom template and boost n = 2.5. (TIF) [file pcbi.1005446.s001.tif]

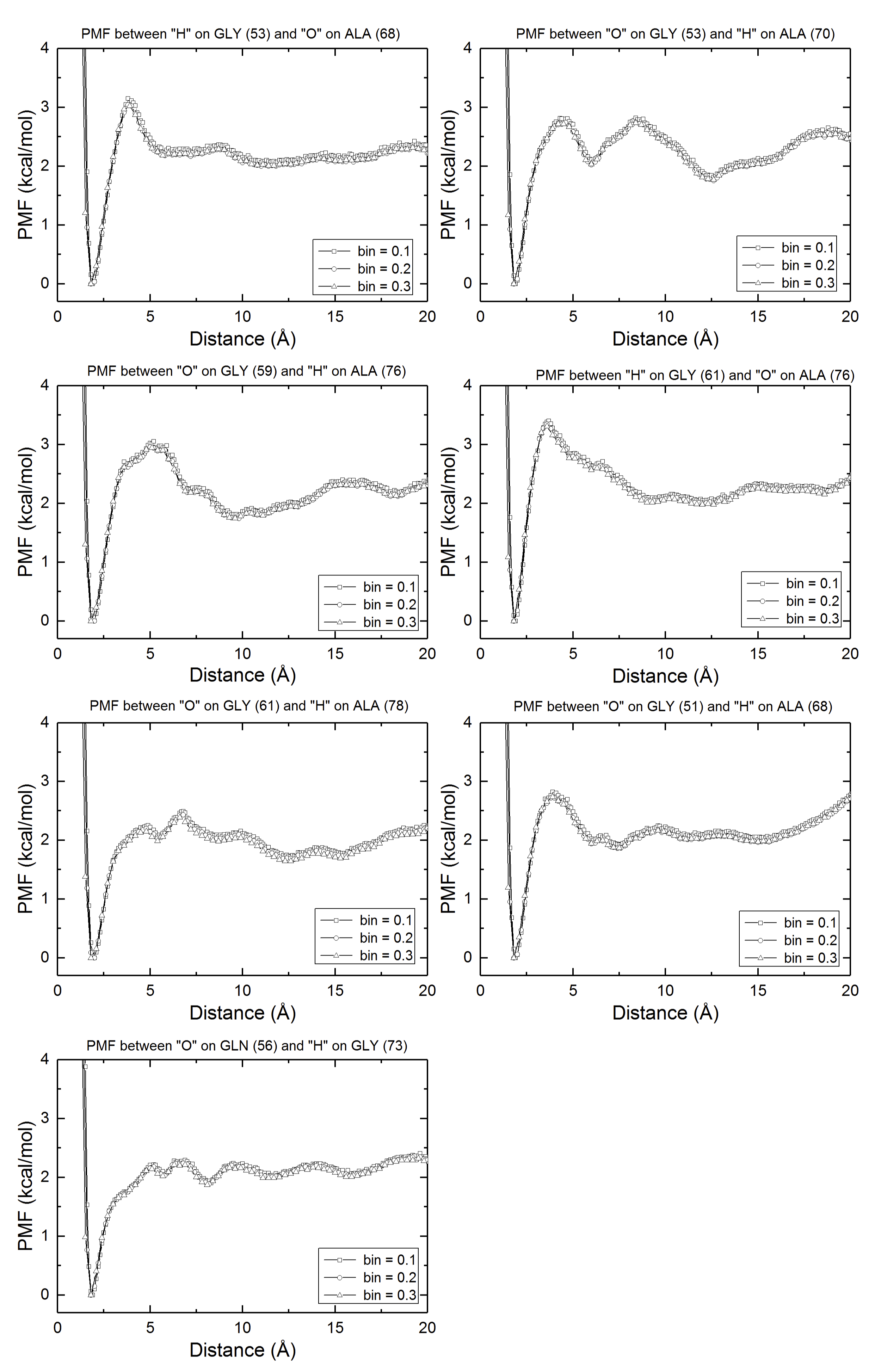

Supplement: S2 Fig — This is for the second set of aMD simulations using boost potential n = 2 and partially-fixed bottom template. (TIF) [file pcbi.1005446.s002.tif]

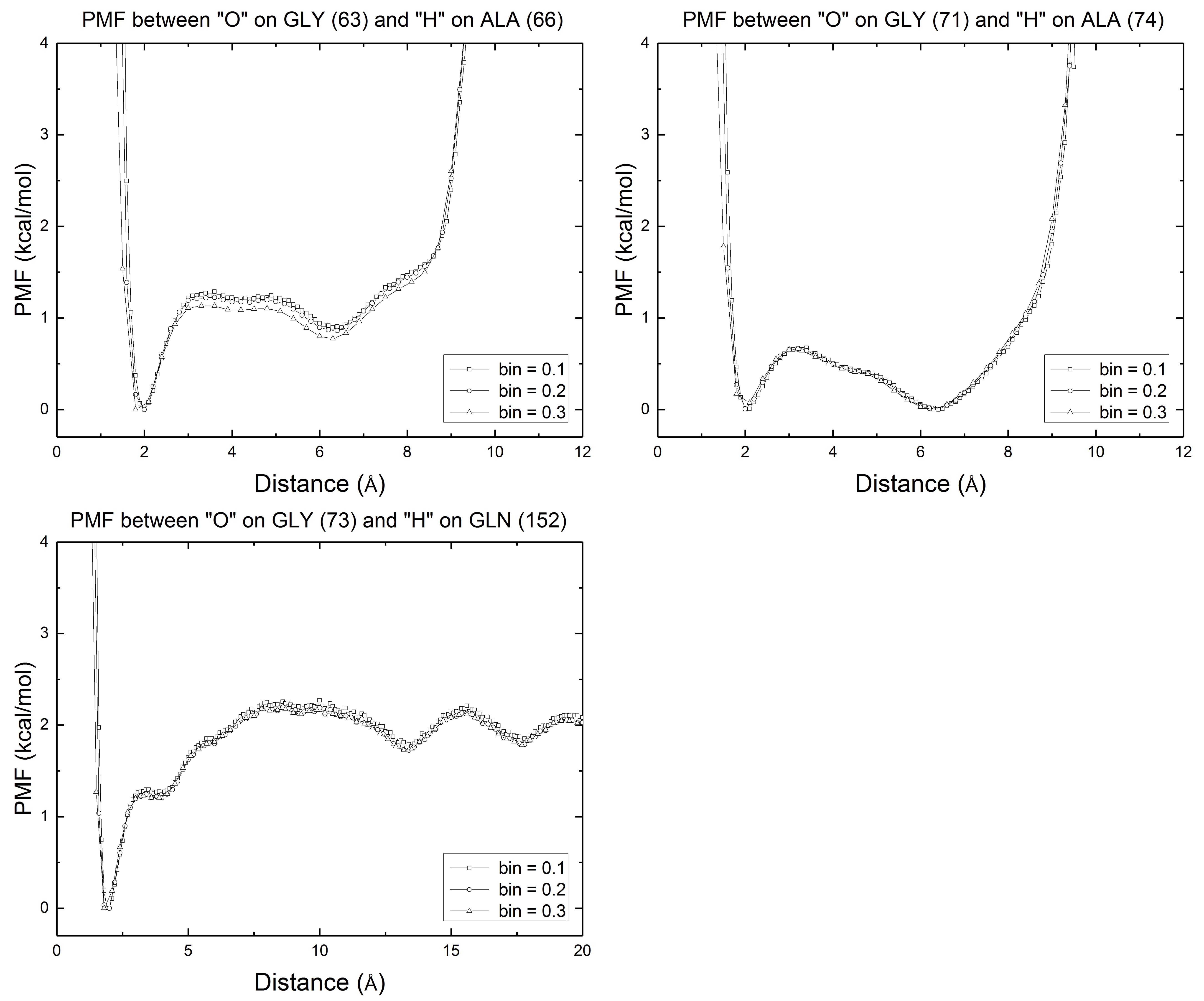

Supplement: S3 Fig — This is for the second set of aMD simulations using boost potential n = 2 and partially-fixed bottom template. (TIF) [file pcbi.1005446.s003.tif]
